# Supplementary material for: Effectiveness and Safety of Ayurvedic Medicines in Type 2 Diabetes Mellitus Management: A Systematic Review and Meta-Analysis
Source: Front Pharmacol. 2022 Jun 8;13:821810. doi: 10.3389/fphar.2022.821810 (PMC9213670; doi:10.3389/fphar.2022.821810)

Study or Subgroup	Trigonella foenum-graecum Mean	SD	Total	Comparator Mean	SD	Total	Weight	Mean Difference IV, Random, 95% CI
**1.1.1 South Asia**								
Gupta 2001	7.54	0.9	12	8.14	1.1	13	6.7%	−0.60 [−1.39, 0.19]
Ansari 2011	8.4	1.9	97	8.68	1.6	92	11.7%	−0.28 [−0.78, 0.22]
Suchitra 2015	7.71	3.91	30	7.8	0.57	30	2.6%	−0.09 [−1.50, 1.32]
Kaur 2016	7.54	3.91	30	7.46	0.57	30	2.6%	0.08 [−1.33, 1.49]
Singh 2016	6.96	0.84	20	7.28	1.06	20	9.7%	−0.32 [−0.91, 0.27]
Ranade 2017	6.94	2.94	30	7.4	2.69	30	2.6%	−0.46 [−1.89, 0.97]
Kandhare 2018	7.25	1.24	58	7.74	1.3	53	12.3%	−0.49 [−0.96, −0.02]
Hota 2019	5.16	3.55	102	5.65	3.45	102	5.0%	−0.49 [−1.45, 0.47]
Rashid 2019	6.23	0.58	32	7.18	0.17	32	19.8%	−0.95 [−1.16, −0.74]
**Subtotal (95% CI)**			**411**			**402**	**72.9%**	**−0.55 [−0.81, −0.28]**
Heterogeneity: $Tau^2 = 0.05$; $Chi^2 = 12.70$, df = 8 (P = 0.12); $I^2 = 37\%$								
Test for overall effect: Z = 4.00 (P < 0.0001)								
**1.1.2 Others**								
Lu 2008	6.56	0.95	46	8.2	1.73	23	7.1%	−1.64 [−2.40, −0.88]
Rafraf 2014	7.74	1.65	44	8.2	1.95	44	7.1%	−0.46 [−1.21, 0.29]
Hassani 2019	7.44	1.27	62	7.96	1.29	63	12.9%	−0.52 [−0.97, −0.07]
**Subtotal (95% CI)**			**152**			**130**	**27.1%**	**−0.84 [−1.53, −0.15]**
Heterogeneity: $Tau^2 = 0.26$; $Chi^2 = 6.86$, df = 2 (P = 0.03); $I^2 = 71\%$								
Test for overall effect: Z = 2.38 (P = 0.02)								
**Total (95% CI)**			**563**			**532**	**100.0%**	**−0.60 [−0.85, −0.36]**
Heterogeneity: $Tau^2 = 0.07$; $Chi^2 = 19.56$, df = 11 (P = 0.05); $I^2 = 44\%$								
Test for overall effect: Z = 4.90 (P < 0.00001)								
Test for subgroup differences: $Chi^2 = 0.60$, df = 1 (P = 0.44), $I^2 = 0\%$								

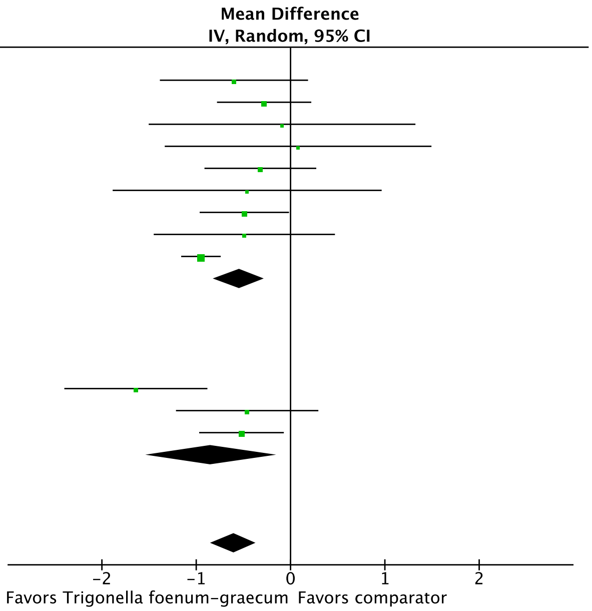

Supplement: Supplementary file 1 [file DataSheet1.zip › Supplementary Material/Forest and Funnel Plots/Trigonella foenum-graecum/Subgroup analyses - country/Trigonella foenum-graecum_HbA1c_country subgroup analysis.pdf]
